# Body size, spontaneous activity and thermogenesis effects on energy expenditure: an introduction to a topic on energy metabolism

**DOI:** 10.3389/fphys.2013.00301

**Published:** 2013-10-17

**Authors:** Patrick C. Even

**Affiliations:** UMR914 Nutrition Physiology and Ingestive Behavior, AgroParisTechINRA, Paris, France

**Keywords:** energy expenditure, indirect calorimetry, body size, body composition, physical activity, brown adipose tissue, metabolic phenotyping, thermogenesis

Life is sustained by the extraction of energy from nutrients. The mechanism is oxidation of the energy-containing macronutrients in food: carbohydrates, lipids and proteins. This result in rates of oxygen consumption and carbon dioxide release closely proportional to the energy extracted from the nutrients. So in effect, measuring respiratory exchanges is measuring life itself.

The measurement of whole body energy expenditure (EE) and substrate utilization by continuous recording of oxygen consumption, carbon dioxide production and when required nitrogen and methane excretion is based on techniques that matured early in the previous century thanks to pioneering work led by researchers such as Rubner, Lusk and Benedict. For those interested in an historical perspective on the concepts of respiration and calorimetry, I strongly suggest reading the richly illustrated review of Frankenfield (2010).

Indirect calorimetry reveals the overall integration of the metabolic pathways controlling energy fluxes and partitioning, and informs if an observed or provoked alteration at the cellular or organ level bears significant consequences at the whole body level. This technique, although appearing simple in its principles, is in fact very sensitive to methodological and conceptual errors, and so requires great care to be correctly used and interpreted. It also raises different problems when applied to humans or to small laboratory rodents. Many technical reviews have been published on this subject, discussing in both animals and humans the apparatus design (Jensen et al., 2001; Kaiyala and Ramsay, 2010; Melanson et al., 2010; Even and Nadkarni, 2012), data processing (Arch et al., 2006; Compher et al., 2006; Schoffelen and Westerterp, 2008; Tschop et al., 2012) and possible limitations of the technique (Walsberg and Hoffman, 2005). Contributors to this topic have focused on three main components of EE susceptible to affect measurements and interpretation of the data; body size, spontaneous activity and thermogenesis.

Whole body EE must be properly adjusted for body size and composition to avoid incorrect interpretations. This is because tissues and organs have different specific metabolic rates, and more globally fat free mass has a larger influence on EE than fat mass (Elia, 1992). This has been extensively discussed by John Arch (Arch and Trayhurn, 2013), plus Anja Bosy-Westphal has reviewed the main strategies used in humans to adjust EE to body size, proposing a new approach to improve adjustment of EE between subjects with wide differences in percent fat mass (Bosy-Westphal et al., 2013). In his review on measuring energy metabolism in the mouse, John Speakman considered the various attempts made to deal with this question in the mouse model (Speakman, 2013).

One main source of variability in total EE is related to the amount of spontaneous activity. It has been a challenge for years to precisely measure it and then compute the consequent energy expenditure. The technical problems may be very different depending on the subject (human vs. animal) and experimental conditions (chambers vs. field measurements). On this topic, Dr Westerterp discussed methods for measurement, determinants and effects of physical activity in humans. He also focused on the interest of the doubly labeled water technique as a field indirect calorimetry method to assess physical activity in humans (Westerterp, 2013). Dr Sarafian (from the laboratory of Dr Dulloo) described a new approach to perform standardized tests for assessing human variability in the energy cost of low-intensity isometric work that is comparable to daily life activities (Sarafian et al., 2013). Etienne Labussière described the method used in his laboratory to deal with this question in large farm animals such as white pigs (Labussiere et al., 2013), and Jan B van Klinken described the most advanced procedures for rats and mice and compared their robustness in relation to the frequency of data acquisition and quality of the activity signal (van Klinken et al., 2013). In his review, John Speakman developed an extensive section on physical activity in which he discussed the various issues related to this question (including treadmill running) and surveyed the most significant published works (Speakman, 2013).

Brown adipose tissue (BAT) is essential in small rodents to maintain body temperature. Since thermoneutrality occurs at 28–32°C, BAT is already very active at the 21°C ambient temperature in most animal facilities. This can increase resting EE in mice up to 2–3 fold when singly housed (Selman et al., 2001) (see also Speakman, 2013) or by 60% when housed in groups with isolative bedding (Cannon and Nedergaard, 2009). In the rat, decreasing temperature from 30 to 20°C increases resting EE by 50% (Evans et al., 2005). Understanding the effect of BAT is thus essential for mouse but also for rat metabolic phenotyping. In humans, until recently it was considered that BAT disappeared rapidly after birth. However, significant amounts of active BAT in humans have been revealed by positron emission tomography (Nedergaard et al., 2007). Acute exposure to cold stimulated and propranolol treatment inhibited activity of these depots (Nedergaard et al., 2010). Thus in a context where no safe and effective drugs are available to treat obesity, this observation has motivated researchers into considering the possibility that stimulating BAT might be effective in treating obesity and its associated metabolic disorders (Bartelt et al., 2011). Sam Virtue has discussed the practical considerations of methods for analyzing BAT tissue function in rodents, including the use of indirect calorimetry and other more simple measurements such as pair feeding, BAT lipid content and protein markers (Virtue and Vidal-Puig, 2013), and Jonathan Arch has discussed the detection of thermogenesis in rodents in response to anti-obesity drugs and genetic modification. He asserts that a proper analysis of the thermogenic response to genetic modifications or pharmacological compounds is essential to decide whether it is worth seeking drugs potentially useful for obesity treatment (Arch and Trayhurn, 2013). Both also reminded us that thermogenesis may be stimulated outside BAT, and drew our attention to the risk of confounding effects on thermogenesis with those on locomotor activity or body composition.

There is much more in each of the articles on this topic than can be commented on in this short introduction. I hope that the readers will go through the articles where they will find a lot of information to better master the possibilities and understand the limitations of measuring EE in humans and animals.
